# Practice and technique of using face mask amongst adults in the community: a cross-sectional descriptive study

**DOI:** 10.1186/s12889-020-09087-5

**Published:** 2020-06-16

**Authors:** Linda Yin-king Lee, Evangeline Pui-wah Lam, Chiu-kiu Chan, Sum-yi Chan, Man-ki Chiu, Wing-hei Chong, Kin-wai Chu, Man-sze Hon, Lok-ki Kwan, Kit-lam Tsang, Siu-lai Tsoi, Chung-wai Wu

**Affiliations:** School of Nursing and Health Studies, The Open University of Hong Kong, Ho Man Tin, Kowloon, Hong Kong

**Keywords:** Face mask, Hand hygiene, Respiratory tract infections, Communicable disease control, Community health

## Abstract

**Background:**

The proper use of face mask comprises the correct practice and wearing technique and is important in preventing the spread of respiratory infections. Previous studies have addressed only the aspect of practice and failed to provide a detailed account of face mask usage amongst community-based populations. This study examined the practice and technique of using face mask amongst adults.

**Methods:**

A cross-sectional descriptive design was adopted. A quota sample of 1500 adults was recruited in Hong Kong during a nonepidemic state between January and February 2017. The participants’ practice of using face mask in five given situations was assessed using a questionnaire. Their technique in using face mask, including 12 steps, was assessed using an observation checklist. Statistical tests were used to compare the differences in practice and technique amongst adults of different gender and age groups.

**Results:**

Findings revealed that the performance of the participants in both categories was unsatisfactory. In terms of practice, less than one-fifth of the participants reported that they always wore face mask when taking care of family members with fever (14.7%) or respiratory infections (19.5%). Male adults and those aged 55–64 reported low frequency in using face mask during required situations. In terms of technique, none of the participants performed all the required steps in using face mask correctly. More than 90% of the participants did not perform hand hygiene before putting on (91.5%), taking off (97.3%), or after disposing (91.5%) face mask. Adults aged 55 and above performed poorer than adults in the younger age groups.

**Conclusion:**

Compared with previous findings obtained during an epidemic, the performance of the participants during a nonepidemic state was less satisfactory. The possibility of developing fatigue after exposure to repeated epidemics was discussed. This study contributes to a comprehensive understanding of the use of face mask in a community and reveals the underperformed areas. Effort is required to enhance the proper practice of using face mask, convey the message that hand hygiene is an essential step in wearing and taking off a face mask and increase the public’s general concern in the value of using face mask.

## Background

A face mask is a loose-fitting and single-use device that covers the nose, mouth and chin. It provides a physical barrier against potentially infectious droplets and is a simple and low-cost nonpharmaceutical individual intervention for protecting oneself and preventing the spread of respiratory infections [1]. Health organisations worldwide recommend the use of face masks to prevent the spread of respiratory infections [1–4].

A face mask should be used correctly to achieve the desired effect. Incorrect usage may increase, instead of decrease, the spread of respiratory infections [5]. The proper use of face mask comprises the correct practice and wearing technique. An assessment on these two aspects can provide pertinent information for the development of health promotion strategies to improve the effectiveness of using face mask.

### Practice of using face mask

The practice of using face mask can be observed in five situations, namely, when (1) taking care of family members with fever, (2) taking care of family members with respiratory infections, (3) visiting clinics during peak season or a flu pandemic, (4) visiting hospitals during peak season or a flu pandemic, and (5) having respiratory symptoms [2]. In the first four situations, people generally wear face mask to protect themselves. In the last situation, people wear face mask to protect others. In circumstances where a respiratory infection has widely spread in the community and can be spread whilst asymptomatic, e.g. in the case of COVID-19, people wear face masks in the first four situations, with an aim to protect others as well.

When caregivers take care of family members with fever or respiratory infections, they risk being infected. Thus, caregivers are recommended to use face mask to protect themselves [2]. However, no study has been conducted on the practice of wearing face mask when taking care of family members with fever or respiratory infections.

When people visit clinics or hospitals during peak season or a flu pandemic, wearing a face mask can protect healthy people from contracting respiratory infections or prevent infected people from spreading diseases. A study conducted at a clinic in Hong Kong revealed that a high percentage of outpatients and their caregivers are likely to wear face mask when visiting clinics to protect others (91.8%) and protect oneself (81.9%) [6]. However, the study was conducted in one clinic, and the sample size of 399 was small; thus, the findings may not truly reflect the public situation. In relation to wearing face mask when visiting hospitals, no study on public practice has been found.

When people have respiratory symptoms, their respiratory droplets usually contain pathogenic microorganisms. If they do not cover their mouth and nose with a face mask, the transmission of respiratory infections is likely to occur. In Hong Kong, the prevalence of wearing face mask amongst adults that have respiratory symptoms during respiratory epidemics was high at 90.0% in 2003 [7], 92.4% in 2005 [8] and 88.7% in 2009 [9]. These high rates are understandable because the chance of spreading respiratory infections is high during an epidemic, when people tend to be more alert and have a high rate of compliance with preventive measures. To understand people’s reactions during a nonepidemic state, further research is needed.

Studies have also examined the association between personal characteristics and the practice of using face mask. Two studies in Hong Kong found that female adults are more likely to wear face mask to protect themselves from respiratory infections; the rate was 66.9% during the outbreak of Severe Acute Respiratory Syndrome (SARS) in 2003 [10] and 92.6% during the early phase of the H1N1 influenza epidemic in 2009 [9]. However, other studies in Australia [11] and Taiwan [12] failed to reveal gender differences in the practice of using face mask. These findings should be interpreted carefully. Differences in findings amongst studies may be due to the change of attitudes over time and cultural differences amongst countries. Moreover, differences in research methodologies may have contributed to the outcome. One study used a relatively small sample size (*n* = 352) [12], thereby reducing the representativeness of its sample. Three studies used telephone survey to collect data [9–11]. Compared with face-to-face interview, a telephone survey is believed to impose extra difficulty for researchers to validate the findings from participants [13]. Thus, studies using other data collection methods, such as face-to-face interview, can provide more valid findings to complement existing findings.

Several studies have examined the association between age and the practice of using face mask and reported inconsistent results. In Hong Kong, middle-aged adults aged 35–60 [8], 45–60 [14] and 50–59 [10] were more likely to wear face masks, with rates of 78.9, 44.4 and 68.2%, respectively. Conversely, the elderly in Japan (aged 60–69) [15] and Australia (aged 65–74) [11] were more likely to wear face mask, and the percentages were found to be 43.6% and > 60%, respectively. However, the use of telephone [8, 10, 11, 14] and online surveys [15] in these studies impeded the assessment of the participants’ age. Therefore, the validity of results was limited.

### Technique of using face mask

The technique of using face mask refers to the manner of wearing and taking off a face mask [16]. The proper technique of wearing a face mask includes the following eight steps: (1) Perform hand hygiene before wearing the face mask. (2) Choose the appropriate size of face mask. (3) Ensure that the coloured side of the face mask is facing outward (for coloured face masks) or that the side with folds is facing downward and outward (for uncoloured face masks). (4) Ensure that the part with metal strip is on the upper side. (5) Position the elastic bands or strings properly. (6) Press firmly on the metal strip so that it follows the shape of the bridge of the nose and face. (7) Extend the face mask to cover the nose, mouth and chin. (8) Avoid touching the face mask once secured on the face or perform hand hygiene before and after touching the face mask. The proper technique of taking off a face mask includes the following four steps: (1) Perform hand hygiene before taking off the face mask. (2) Touch only the elastic bands. (3) Dispose of the used face mask in a plastic or paper bag or a lidded rubbish bin. (4) Perform hand hygiene after disposing of the face mask [16].

Although the wearing and taking off of face mask require several steps, previous research only focused on a few steps in wearing face mask. A study used a questionnaire to assess outpatients and their caregivers’ knowledge on three steps, including what the face mask covers, where the metal strip should be placed and which side should face outward. Although 88.4% of the participants believed they knew the correct steps for wearing a face mask, only 52.0% answered the questions correctly [6]. This study assessed the technique subjectively by only asking participants whether they knew the correct steps; thus, illustrating the actual performance of the participants was difficult. This limitation reduces the validity of findings.

In summary, the focus of several studies has been identified. Regarding the practice of using face mask, previous studies revealed a compliance rate of as high as 80% when participants visited clinics or hospitals during peak season or a flu pandemic or when they had respiratory symptoms. However, no study has investigated the practice of wearing face mask in the five situations mentioned above. Previous studies were mainly conducted during an epidemic; hence, people’s practice during a nonepidemic state remains uncertain. Regarding the technique of using face mask, previous studies mostly focused on the wearing technique and mainly used a self-reporting method to assess participant technique. Therefore, the validity of previous findings in reflecting the actual performance of participants is doubtful. To date, public knowledge on the practice and technique of using face mask and its association with personal characteristics remains inconclusive. Adults account for a substantial proportion of the population in most communities. Therefore, the way they use a face mask is an essential issue in protecting community health. A study that assesses the practice and technique of adults when using face mask can provide useful information to the planning and delivery of health promotion activities in the community.

## Methods

### Objectives and design

The objectives of this study were to (1) examine the practice and technique of adults when using face mask and (2) compare the differences amongst adults of different genders and age groups in terms of their practice and technique of using face mask. This study adopted a cross-sectional descriptive design, which supported the researchers in assessing and describing the performance of the participants at a specific time point (i.e. during a nonepidemic state) rather than over a period. In this manner, the study objectives were achieved. The face mask used in this study was an earloop-type surgical mask which is commonly used by the public [16].

### Setting and sample

A quota sample of 1500 adults was recruited from public areas in Hong Kong between January and February 2017. No respiratory epidemic occurred during this period. This study could not use power analysis to determine the sample size because previous findings on gender and age differences in terms of the practice and technique of using face mask were inconsistent and inconclusive. Instead, this study considered recruiting the maximum number of participants within the available time and resources. In accordance with the principle of ‘the larger the sample, the greater the statistical power’ [17], a large sample could determine true differences between the adult groups.

The sample was stratified by age into seven strata in accordance with the age categories that are commonly adopted for Hong Kong population statistics. The proportion of subjects in each stratum was determined on the basis of the proportion of adults in the Hong Kong population [18]. The intention was to obtain a sample in terms of age population that are close to the Hong Kong population. In this study, probability sampling was not feasible because a sampling frame was unavailable. Despite using a nonprobability sampling method, quota sampling increases the representativeness of the sample [19]. Eligible participants included people aged 18 or above and could communicate in Cantonese (the major dialect in Hong Kong). Adults who reported allergy to face masks or alcohol hand rub were excluded.

### Data collection

The two variables of interest include the practice and technique of using face mask. The practice of using face mask comprises the act of wearing a face mask during required situations [2]. This variable was measured using a questionnaire. The technique of using face mask refers to the manner of wearing and taking off a face mask [16]. This variable was measured using an observation checklist. The content of the questionnaire and the observation checklist was based on the guidelines in ‘Use Mask Properly’, which was developed in accordance with international standards and published for public use in Hong Kong [16].

The questionnaire was composed of five statements, and each statement represented a specific situation that requires wearing a face mask. A five-point Likert scale was provided to the participants to indicate the frequency of their face mask usage in each given situation. ‘Always’ indicated wearing a face mask 10 times within 10 times (score 5). ‘Often’ indicated wearing a face mask 7–9 times within 10 times (score 4). ‘Sometimes’ indicated wearing a face mask 4–6 times within 10 times (score 3). ‘Seldom’ indicated wearing a face mask 1–3 times within 10 times (score 2). ‘Never’ indicated not wearing a face mask within 10 times (score 1). ‘Not applicable’ indicated that the participants did not encounter a situation that requires wearing a face mask. If participants reported ‘Not applicable’ in a specific situation, then the response to that situation was excluded in the analysis. Since the five given situations were unique in nature, it was irrational to sum up participants’ performance in these situations and to develop an overall performance. Therefore, calculating a total score for this questionnaire was not applicable.

The content validity of the questionnaire was evaluated by an expert panel, namely, three nursing academicians with background in infection control and three clinical nurses with at least 10 years of clinical experience. The questionnaire reported a content validity index of 1.0, which indicates high content validity [20]. The stability of the questionnaire was evaluated via test–retest reliability testing. Fifty adults were invited to complete the questionnaire on two separate occasions within a two-week interval. The test–retest reliability coefficient was 0.83, indicating good test–retest reliability [21].

The observation checklist was composed of 12 items. Eight items addressed the technique of wearing a face mask, whereas four items addressed the technique of taking a mask off. Each item represented a specific step in wearing or taking off a face mask. ‘Correct’ (score 1) and ‘Incorrect’ (score 0) were provided as options for the rater to assess how well the participants performed the technique. The total score obtained from these 12 items was calculated. A high score indicated an excellent performance in using face mask. A space next to the checklist was provided to enable the researcher to describe narratively why a technique is incorrect.

The observation checklist underwent psychometric testing, and its content validity was evaluated by the abovementioned expert panel. The checklist reported a content validity index of 1.0, which indicates high content validity [20]. Inter-rater reliability was assessed by asking the two raters to make simultaneous independent observations on the technique of 20 participants. A kappa coefficient of 0.92 was obtained, indicating a high level of inter-rater agreement [22].

Data were collected from public areas in Hong Kong. Participants answered the questionnaire via a face-to-face structured interview. Two trained researchers were assigned to conduct the interviews, thereby minimising the chance of having inconsistency in asking the questions. Participants were then instructed to wear and take off a face mask using provided equipment, namely, coloured earloop-type surgical masks in various sizes, a bottle of alcohol hand rub, a rubbish bin with a lid and a rubbish bin without a lid. The researcher rated the participants’ performance based on the observation checklist. Specific measures were implemented to minimise the Hawthorne effect. Only one researcher was assigned to perform every single observation, thereby minimising the embarrassment of a participant. The researcher assured the participant of the confidentiality of data handling and proper disclosure of research findings, thus relieving the apprehension of the participant.

This study followed the ethical standards specified by the Declaration of Helsinki. Ethical approval was obtained from the Research Ethics Committee of the university in which the researchers were affiliated. The researchers explained the nature of their study to potential participants and informed them of their right to participate and withdraw. Anonymity was ensured by not collecting any personal identifying information. Confidentiality was ensured by not allowing unauthorised persons to gain access to all data. Participants gave informed consent verbally. This is a common practice in face-to-face questionnaire survey. The Research Ethics Committee approved this practice.

### Statistical analyses

Statistical Package for the Social Sciences Version 23.0 was used for data analysis. No missing data were reported. Descriptive statistics was used to describe the participants’ demographic characteristics and their practice and technique in using face mask, whereas inferential statistics was performed to compare group differences. The considerations between using parametric and nonparametric tests depended on the level of measurement and distribution of data.

Nonparametric tests were used to analyse the data of the practice of using face mask because data were presented in an ordinal level. The Mann–Whitney U test was used to compare the differences in the practice of using face mask in each given situation amongst adults of different gender groups. The Kruskal–Wallis test was used to compare the differences in the practice of using face mask in each given situation amongst adults of different age groups. The Bonferroni–Dunn test was used as post-hoc test to perform pairwise multiple group comparisons [23]. In this study, the practice of using face mask was measured on the basis of the participants’ performance in the five given situations which were unique in nature. Summing up the participants’ performance in these situations and to develop an overall performance is irrational. Thus, a comparison between groups was conducted at the item level.

Parametric tests were used to analyse the data of the technique of using face mask because data were presented in a ratio level. Prior to data analysis, normality analyses, including skewness, kurtosis, histogram and probability–probability plot, were performed to confirm the normal distribution of the number of correctly performed items in the technique of using face mask. The study variable was normally distributed and fit for parametric analysis. An independent t-test was used to compare the differences in the technique of using face mask amongst adults of different gender groups. One-way analysis of variance (ANOVA) was used to compare the differences in the technique of using face mask amongst adults of different age groups. The Games–Howell test was used as a post-hoc test to perform pairwise group comparisons [24]. The significance level was < 0.05.

## Results

### Sample characteristics

A total of 1987 adults were approached. Whilst the rest of the approached adults refused to participate, 1500 adults agreed and participated in this study. Half of the participants were female (52.9%). Participants aged 45–54 years old represented the largest age group (21.5%). The age and gender distributions of this sample were comparable with the Hong Kong population in 2011 [18] (Table 1). The 2011 statistics were the most updated population statistics at the time when the study was conducted.

**Table 1:** Demographic characteristics of participants

Demographic characteristic	Study sample (*n* = 1500)		Hong Kong population (> 18 years old) (*n* = 5,999,440)	
	Frequency	Percentage (%)	Frequency	Percentage (%)
Gender				
Male	706	47.1	2,747,743	45.8
Female	794	52.9	3,251,696	54.2
Age				
18–24 years old	157	10.5	626,658	10.5
25–34 years old	271	18.1	1,084,120	18.1
35–44 years old	284	18.9	1,135,285	18.9
45–54 years old	322	21.5	1,289,430	21.5
55–64 years old	231	15.4	922,635	15.4
65–74 years old	116	7.7	464,740	7.7
> 75 years old	119	7.9	476,572	7.9

### Practice of using face mask

Overall, the participants’ performance in the practice aspect was unsatisfactory. Only 114 participants (7.6%) reported that they always wore a face mask in the five required situations. Less than half of the participants (range of 14.7–48.1%) reported that they always wore a face mask under individual required situations (Table 2).

**Table 2:** Overall practice of using face mask

Practice of using face mask	Never	Seldom	Sometimes	Often	Always	Not applicable
	Frequency (%)					Frequency
1. When taking care of family members with fever. (*n* = 1362) ^a^	428 (31.4)	336 (24.7)	219 (16.1)	178 (13.1)	201 (14.7)	138
2. When taking care of family members with respiratory infection. (*n* = 1370) ^a^	368 (26.9)	290 (21.2)	235 (17.1)	210 (15.3)	267 (19.5)	130
3. When visiting clinics during peak season or a flu pandemic. (*n* = 1478) ^a^	167 (11.2)	256 (17.3)	246 (16.6)	281 (19.0)	528 (35.7)	22
4. When visiting hospitals during peak season or a flu pandemic. (*n* = 1463) ^a^	110 (7.5)	168 (11.5)	195 (13.3)	286 (19.6)	704 (48.1)	37
5. When having respiratory symptoms. (n = 1463) ^a^	161 (10.8)	270 (18.2)	332 (22.4)	289 (19.5)	431 (29.1)	17

^a^Participants who reported “Not Applicable” were excluded

The Mann–Whitney U test showed that compared with females, males reported lower frequency in using face mask in the five required situations (Table 3). The Kruskal–Wallis test showed that participants of different age groups reported different practices of using face mask when visiting clinics during peak season or a flu pandemic (χ^2^ = 14.46, *p* < 0.05) and when having respiratory symptoms (χ^2^ = 27.75, *p <* 0.001). Findings from the post-hoc test (Bonferroni–Dunn) with adjusted level of significance (*p* < 0.001) supported that participants aged 55–64 (mean rank = 669.18) had a lower frequency of using face mask in comparison with adults aged 18–24 (mean rank = 850.11) when having respiratory symptoms (*p* < 0.001). However, the other pairs of group comparisons were nonsignificant (Table 3).

**Table 3:** Practice of using face mask between adults in different gender and age groups

Practice of using face mask	Gender			Age group							
	Male	Female	*U*	18–24	25–34	35–44	45–54	55–64	65–74	> 75	*χ*^*2*^
	Mean rank			Mean rank							
1. When taking care of family members with fever. (n = 1362) ^a^	646.86	711.66	208,817.00**	702.03	703.46	705.76	661.54	619.15	679.42	718.11	9.52
2. When taking care of family members with respiratory infection. (n = 1370) ^a^	652.74	713.80	212,563.00**	767.86	703.92	698.49	660.94	649.02	660.26	666.93	11.12
3. When visiting clinics during peak season or a flu pandemic. (n = 1478) ^a^	675.81	796.03	227,830.00***	813.13	739.32	735.94	756.27	692.17	654.24	780.63	14.46* ^b^
4. When visiting hospitals during peak season or a flu pandemic. (n = 1463) ^a^	665.20	791.13	220,667.50***	811.35	738.37	711.45	745.39	700.75	670.04	744.97	11.43
5. When having respiratory symptoms. (n = 1463) ^a^	656.12	817.95	214,101.00***	850.11	731.82	776.83	746.45	669.18	690.79	715.88	27.75***^c^

*p < 0.05, **p < 0.01, ***p < 0.001

^a^Participants who reported “Not Applicable” were excluded

^b^Post hoc analysis using Bonferroni-Dunn test revealed a non-significant result

^c^Post hoc analysis using Bonferroni-Dunn test revealed a significant difference in practice between participants in “18–24 years old” and “55–64 years old”

### Technique of using face mask

The performance of the participants in terms of technique was unsatisfactory. None of the participants could correctly perform all 12 steps. On average, they could correctly perform six out of 12 steps. Majority of the participants did not perform hand hygiene before putting on (91.5%), taking off (97.3%) or after disposing of (91.5%) the face mask. Two-thirds of the participants (63.6%) touched the face mask after securing it. Similarly, two-thirds of the participants (70.3%) touched the body of the face mask instead of the elastic bands whilst taking the face mask off (Table 4).

**Table 4:** Technique of using face mask (*n* = 1500)

Technique of using face mask	Correct	Incorrect	Description of incorrect technique
	Frequency (%)	Frequency (%)	
Wearing a face mask			
1. Perform hand hygiene before wearing the face mask.	128 (8.5)	1372 (91.5)	Participants did not perform hand hygiene before putting on the face mask.
2. Choose the appropriate size of face mask.	1126 (75.1)	374 (24.9)	Participants chose an oversized face mask or chose an undersized face mask.
3. Ensure the colored side of the face mask is facing outwards.	1275 (85.0)	225 (15.0)	Participants placed the colored side of the face mask inwards.
4. Ensure the part with metallic strip is on the upper side.	1427 (95.1)	73 (4.9)	Participants placed the metallic strip lowermost.
5. Position the elastic band properly.	1458 (97.2)	42 (2.8)	Participants positioned the elastic band to the middle of the ear back or curled up the elastic band to the arm of the glasses.
6. Press firmly on the metallic strip to the bridge of nose and face.	742 (49.5)	758 (50.5)	Participants did not mold the metallic strip or molded the metallic strip up to the bridge of the nose only.
7. Extend the face mask to cover mouth, nose and chin.	1262 (84.1)	238 (15.9)	Participants did not extend the face mask or extended the face mask partially to cover the nose and mouth only.
8. Avoid touching the face mask once it is secured.	546 (36.4)	954 (63.6)	Participants touched the face mask while handling the glasses, scratching the face or rubbing the nose.
Taking off a face mask			
9. Perform hand hygiene before taking off the face mask.	40 (2.7)	1460 (97.3)	Participants did not perform hand hygiene before taking off the face mask.
10. Touch only the elastic bands.	445 (29.7)	1055 (70.3)	Participants touched the surface of the face mask or folded the face mask inside the palm.
11. Dispose of the used face mask in a lidded rubbish bin.	603 (40.2)	897 (59.8)	Participants disposed the used face mask in a rubbish bin without a lid.
12. Perform hand hygiene after disposing the face mask.	127 (8.5)	1373 (91.5)	Participants did not perform hand hygiene after disposing the face mask.

The independent t-test did not reveal any difference in the technique of using face mask amongst adults of different genders (*t* = − 1.460, *p* > 0.05) (Table 5). One-way ANOVA showed a significant difference in the technique of using face mask amongst adults of different age groups (*F* = 10.24, *p* < 0.001). Post-hoc analysis using the Games–Howell test for nonequal variances indicated that adults aged 55 and above performed considerably poorer than the younger age groups (Table 5).

**Table 5:** Technique of using face mask between adults in different gender and age groups (*n* = 1500)

Technique of using face mask	Gender			Age group							
	Male	Female	*t*	18–24	25–34	35–44	45–54	55–64	65–74	> 75	*F*
	Mean (SD)			Mean (SD)							
Number of steps performed correctly (Total steps = 12)	6 (1.82)	6 (1.68)	−1.460	6 (1.68)	6 (1.66)	6 (1.70)	6 (1.77)	6 (1.52)	5 (1.86)	5 (1.97)	10.24***^†^

***p < 0.001

^†^Post hoc analysis using the Games-Howell test revealed a significant difference in technique between participants in “25–34 years old” and “55–64 years old” (*p* < 0.001), between participants in “25–34 years old” and “65–74 years old” (*p* < 0.001), between participants in “25–34 years old” and “75 years old or above” (*p* < 0.001), between participants in “35–44 years old” and “55–64 years old” (*p* < 0.001), between participants in “35–44 years old” and “65–74 years old” (*p* < 0.001), between participants in “35–44 years old” and “75 years old or above” (*p* < 0.001), between participants in “45–54 years old” and “55–64 years old” (*p* < 0.001), between participants in “45–54 years old” and “65–74 years old” (*p* < 0.001), as well as between participants in “45–54 years old” and “75 years old or above” (*p* < 0.001)

## Discussion

The findings revealed that adults in Hong Kong during a nonepidemic state were not using face mask properly. A separate discussion on practice and technique is presented in the following sections.

### Unsatisfactory practice of using face mask

Less than one-fifth of the participants reported that they always wore a face mask when taking care of family members with fever (14.7%) or respiratory infections (19.5%). The participants probably thought that wearing a mask is unnecessary for a caregiver who is healthy. In addition, they might not have realised that wearing a face mask could effectively block infectious particles expelled from other people with respiratory diseases [25] and probably worried that other people might misinterpret them as sick and infectious. However, a higher percentage was reported in a previous Hong Kong study in which 46.8% of the public wore a face mask at home for self-protection [6].

Less than half of the participants reported that they always wore a face mask when visiting clinics (35.7%) or hospitals (48.1%) during peak season or a flu pandemic. Despite the low percentage, these two situations reported the highest percentage of participants who always wore face mask in this study. This phenomenon might be induced by the free provision of face masks for visitors in many healthcare institutions, the high prevalence of using face mask amongst healthcare professionals and the awareness of the high risk of disease transmission in clinics and hospitals in Hong Kong. Conversely, a previous study in Hong Kong reported a relatively large percentage of outpatients and caregivers wearing face mask when visiting clinics to protect others (91.8%) and themselves (81.9%) [6].

Only one-third of the participants reported that they always wore a face mask when having respiratory symptoms (29.1%). Their practice was considered unsatisfactory. Immediately after the epidemic of SARS in 2003, the people of Hong Kong have developed a consensus on wearing a face mask when having respiratory symptoms [9]. However, this practice revealed a downward trend over the years. The prevalence of wearing face mask amongst adults with respiratory symptoms was high, with a percentage of 90.0% during the SARS epidemic in 2003 [7], 92.4% during an anticipated H5N1 epidemic in 2005 [8] and 88.75% during the early phase of the H1N1 influenza epidemic in 2009 [9]. However, the rate was reduced to 39.0% during the pandemic preparation for H7N9 influenza in 2014 [26] and further to 29.1% during a nonepidemic state in 2017 (the present study).

The overall decrease in the practice of using face mask over the last decade is possibly related to the decrease in the severity of respiratory infections that occur in Hong Kong. In 2003, around 300 people in Hong Kong died due to the SARS pandemic [27]. In 2014, no death was reported in the H7N9 influenza pandemic [28]. A low fatality rate from a respiratory pandemic likely results in less compliance with preventive measures, such as wearing face masks [11]. Moreover, people may have developed fatigue after exposure to repeated epidemics. In addition, the nature of the health atmosphere might have contributed to the outcome. At the time of the study in Hong Kong, respiratory infection was a generic health topic rather than a focused one. Health education on wearing face masks was generally conducted through pamphlets and videos that were delivered in clinics, hospitals and online platforms. Thus, people tend to hold inactive attitudes and adopt preventive practices less [26]. Our study revealed an alarming finding that the adults’ practice of using face mask during a nonepidemic state is unsatisfactory; this issue deserves further attention.

### Unsatisfactory technique of using face mask amongst adults

This study revealed that the performance of the participants in terms of technique was unsatisfactory. Overall, they performed only six out of the 12 required steps correctly. Moreover, their performance in each of the steps varied greatly. Findings suggested that the technique of using face mask cannot be broadly interpreted as a single skill. People can perform some steps correctly but perform other steps incorrectly. This study reveals the performance of the participants in each step and contributes to the enrichment of our understanding in this area.

#### When wearing a face mask

An earlier study found that 88.4% of the participants believed that they knew the proper steps for wearing a face mask, but only 52.0% answered the procedural questions correctly [6]. The current study further revealed that only 2.3% of the participants correctly performed all eight steps for wearing a face mask. Belief, knowledge and behaviour are different concepts, and their relationships are only weak to moderate [6]. Holding belief and knowledge on a health behaviour does not imply that one performs that health behaviour. The large difference in the reported percentages between these studies suggests that simply spending effort to promote belief and increase knowledge of a health behaviour for a population is unlikely to result in a behavioural change. Efforts should focus on directly teaching the required skill and modifying factors that have considerable effect on behavioural change.

The performance of the participants in the eight steps for wearing a face mask varied greatly, as 97.2% of the participants positioned the elastic bands properly, but only 8.5% performed hand hygiene before putting on the face mask. The least performed step was related to hand hygiene. Specifically, performing hand hygiene before putting on a face mask can reduce inadvertent contact between the face and contaminated hands [29]. Participants were possibly unaware that performing hand hygiene was a step before wearing a face mask. The lack of hand hygiene facilities in public areas, such as clinics and hospitals, might have also contributed to this unsatisfactory result.

#### When taking off a face mask

The technique in taking off a face mask is a neglected research area. Findings revealed that only 0.2% of the participants correctly performed the four steps in taking off a face mask. Possibly, people tend to overlook the value of the proper technique in taking off a face mask. Performing the proper technique in removing a face mask is essential to controlling the spread of respiratory infections. Similar to the phenomenon of wearing a face mask, the least performed steps in taking off a face mask were related to hand hygiene. The abovementioned factors that lead adults not to perform hand hygiene before wearing a face mask might have contributed to the poor results of taking off a face mask as well.

Face mask has relatively simple designs, leading many people to assume that they know how to use it [6]. This assumption can possibly reduce people’s desire to learn the proper way of using face mask. Moreover, face mask designs vary amongst manufacturers and are confusing to users. For example, several face masks have a coloured side, which is supposed to face outward, whereas others have the same colour, often white, on both sides. This problem is further compromised as most of the packaging of face masks do not provide clear instructions regarding its proper use. These factors might have further contributed to the unsatisfactory performance reported in this study.

### Practice and technique of using face mask amongst adults of different gender and age groups

Results revealed that the practice of wearing face masks amongst male adults was poorer than that amongst females. This study supports earlier studies, which reported that male adults were less likely to wear face mask [9, 10]. Generally, male adults hold beliefs related to masculinity and perceive themselves as strong with a lower chance of acquiring illnesses. They are less likely to engage in health-related preventive measures. Conversely, female adults, who are more likely to adopt a caregiving role, may consider themselves to have a high risk for illnesses. Thus, females are more likely to adopt health-related preventive measures [30]. Although a previous study reported a nonsignificant difference between genders in performing respiratory preventive behaviour, the performance of the participants might have been overridden by the health threat of the anticipated H5N1 epidemic when the study was conducted [8].

Despite demonstrating a significant difference in their practice of using face masks, male and female adults did not demonstrate any difference in their technique in using face masks. Findings indicated that practice and technique are different concepts. Assuming that people who maintain a satisfactory practice in using face mask can also demonstrate a satisfactory technique in using face mask is illogical. Therefore, teaching and reinforcing the public about the proper technique in using face mask, even to people with good practice, are necessary approaches.

Findings revealed that compared with younger adults (aged 18–24), older adults (aged 55–64) were less likely to wear face masks when having respiratory symptoms. Similarly, older adults (aged 55 or above) demonstrated a more improper technique of using face mask in comparison with the younger ones (aged 25–54). Presumably, older adults do not use face mask because they have less resources and reduced capability to buy face masks and commonly depend on others to obtain face mask. Moreover, older adults have a low chance and capability to receive health promotion information. The unsatisfactory practice and technique in using face mask amongst the older adults warrants attention because older adults are more likely to acquire respiratory infections and suffer from severe complications.

### Strengths and limitations of the study

This study stratified the samples on the basis of the age proportion of adults in Hong Kong. Compared with convenience sampling, using this method ensures the recruitment of a better representative sample. Moreover, this study adopted structured observation to assess the participants’ technique in using face mask. Structured observation is advantageous over the self-reporting method in terms of assessing the participants’ actual performance of the technique. These two approaches greatly enhanced the generalisability of the study results. Although this study attempted to implement measures to minimise Hawthorne effect, reflecting the participants’ actual performance fully remained impossible. Even though the nature of the incorrect technique performed by the participants was not analysed, the effect of such limitation on the generalisability of the results is insignificant.

### Recommendations

Future researchers should examine the factors that affect the practice and technique of adults in using face mask. Understanding the effect of these associated factors can direct and strengthen health promotion strategies.

Health care professionals should focus on unsatisfactory practice and technique in their health promotion activities. In addition to presenting the proper practice and technique of using face mask, reminding people about the frequently omitted and incorrectly performed steps is essential. Especially, the message that hand hygiene is an essential step in wearing and taking off a face mask must be conveyed. Moreover, concrete measures are required to strengthen proper practice and technique of using face mask amongst male and older adults who demonstrated less desirable performance compared with their counterparts. For example, face masks can have masculine designs to address the concern of male adults who feel less manly when wearing face mask. Health education materials can also adopt large fonts and simple diagrams to present the message. In this manner, older adults with visual and language limitations can use these materials without great difficulty. In response to the occurrence of public fatigue due to repeated exposure to epidemics, intense effort to increase the public’s general concern in the value of using face mask is crucial to sustain previous efforts and prevent the current practice from further deterioration [11, 31].

Manufacturers should redesign the package of face masks and incorporate instructions regarding the proper practice and correct technique of using face mask. These instructions can remind people and reinforce their action immediately before they use face mask.

The government should develop policies that can enhance the proper use of face mask. A policy requiring all clinics, hospitals and even public areas to provide free face masks and accessible hand hygiene facilities to visitors can increase public compliance with the proper practice. Moreover, a policy requiring face mask manufacturers to incorporate instructions on the packaging can remind and reinforce users of the proper technique when they use face mask. In terms of social welfare, free face masks can be provided to households with older adults who do not have the resources and capability to buy face masks. This strategy involves the concerted efforts of various social welfare and volunteer groups.

## Conclusion

The proper use of face mask is essential to minimising the spread of respiratory infections in a community. This study examined the practice and technique of using face mask amongst adults during a nonepidemic state and revealed their unsatisfactory performance. An all-out effort of the researchers, health care professionals, manufacturers and the government will increase people’s awareness of the proper practice and technique and enhance the performance of adults in using face mask. Although the study was conducted in Hong Kong, findings and implications are relevant to other countries.

## Data Availability

The data collection instruments and the datasets used during the current study are available from the corresponding author on reasonable request.

## Abbreviations

ANOVA: One-way analysis of variance
SARS: Severe Acute Respiratory Syndrome
